# A Cause-Effect Relationship of Infliximab and Pulmonary Embolism: A Case Report

**DOI:** 10.7759/cureus.9615

**Published:** 2020-08-08

**Authors:** Areeg Bala, Mahmoud Barbarawi, Shima Sidahmed, Ashok Kumar Kanugula, Ghassan Bachuwa

**Affiliations:** 1 Internal Medicine, Hurley Medical Center/Michigan State University, Flint, USA

**Keywords:** infliximab, pulmonary embolism, coagulopathy, subsegmental pe, remicade, crohn disease

## Abstract

Biological monoclonal antibodies such as infliximab have significantly remodeled inflammatory bowel disease's treatment course. Despite multiple side effects reported with infliximab, this medication has shown to be robust and practical. There is a paucity of cases reporting venous thromboembolism (VTE) occurrence during the treatment with infliximab. Although such an association's exact mechanism is still not precise, we should be aware of the drug thromboembolic aptitude. Close attention should be given to patients who started infliximab infusion for any symptoms of pulmonary embolism or deep vein thrombosis.

## Introduction

Infliximab has been used for many years as a primary treatment of autoimmune disease, most commonly for inflammatory bowel disease (Crohn's disease and ulcerative colitis), rheumatoid arthritis, and seronegative arthritis [1]. Common side effects associated with infliximab are due to immune suppression as it is a monoclonal biological antibody. By suppressing immunity, susceptibility to serious infections arises, for example, reactivation of hepatitis B and tuberculosis [2]. Other side effects include thrombocytopenia and leukopenia [3]. Few and very rare side effects were reported sporadically, like drug-induced lupus, positive antinuclear antibody (ANA), and pulmonary embolism [4]. Our case represents a rare occurrence of pulmonary embolism in a patient treated with infliximab.

## Case presentation

**Figure 1:**
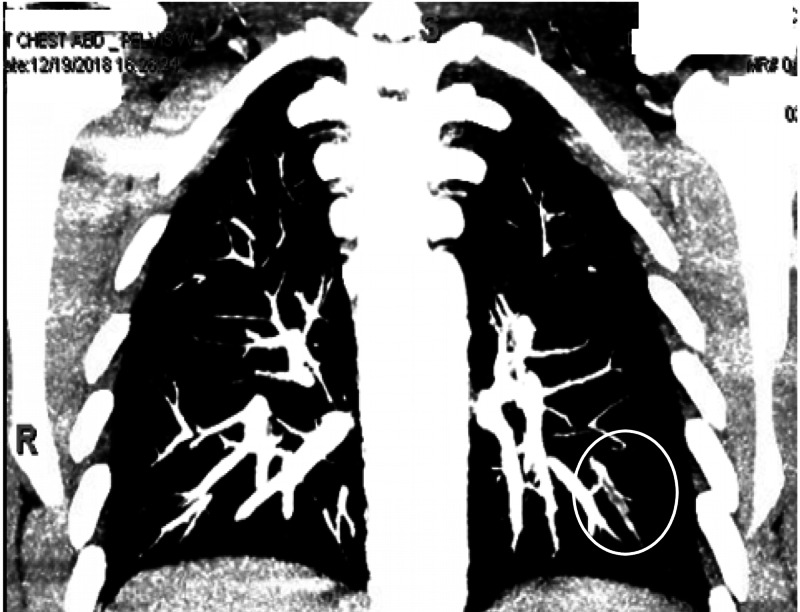
CT scan of the chest with contrast (coronal view) showing a filling defect in the left lower lobe segmental and subsegmental pulmonary arteries consistent with pulmonary embolism.

**Figure 2:**
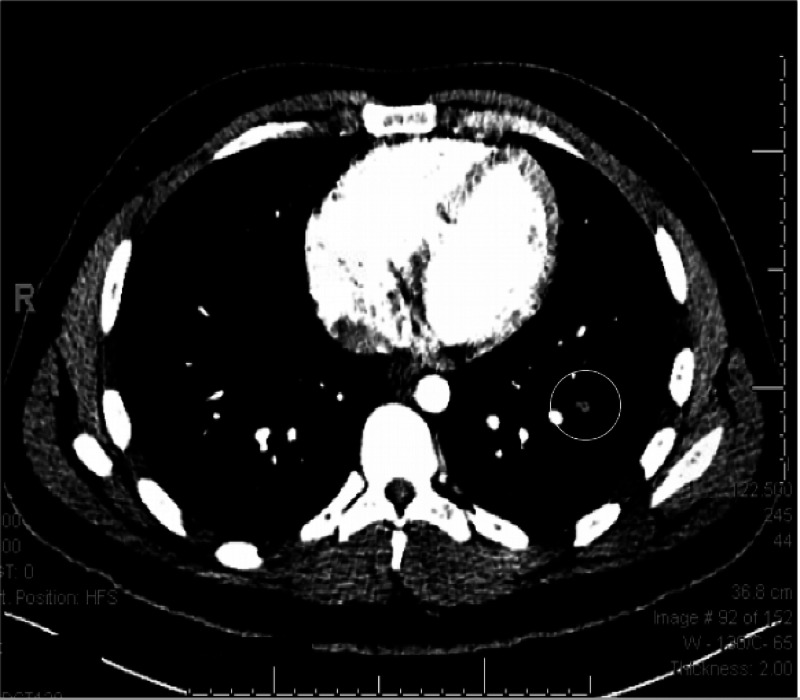
CT scan of the chest with contrast (axial view) showing a filling defect in the left lower lobe segmental and subsegmental pulmonary arteries consistent with pulmonary embolism.

A 20-year-old African American male presented with a past medical history of type 1 diabetes mellitus and a recently diagnosed Crohn's disease six months before presentation and was actively on mesalamine and infliximab infusions. His last injection was one week before the presentation. He presented to the emergency department with a complaint of chest pain and back pain for one week. His chest pain was dull, diffuse, continuous, increased with respiration, and associated with shortness of breath. On examination, his vital signs showed tachycardia with a heart rate of 139 beats per minute, respiratory rate 18 breaths per minute, blood pressure 112/52 mm Hg, and temperature of 37.4° C. Laboratory investigations included EKG with sinus tachycardia and a chest X-ray that failed to reveal any pulmonary process. The patient had a D-dimer elevated at 2,804.80 mcg/mL. His computerized tomography (CT) of the chest revealed pulmonary embolism involving the left lower lobe segmental and subsegmental vessels (Figures 1, 2). Notably, the patient had no previous history of smoking, recent immobilization, surgery, travel, or any personal or family history of clotting disorders. Intravenous heparin was initiated for provoked pulmonary embolism. He was released home on a subcutaneous anticoagulant for three months. A subcutaneous anticoagulant was preferred over an oral anticoagulant in this case due to the difficulties absorbing oral anticoagulants given the patient's disease. The patient was instructed to follow up with his gastroenterologist to navigate an alternative treatment for his Crohn's disease.

## Discussion

Patients with inflammatory bowel disease (IBD) have a higher incidence of venous thromboembolism (VTE); they have two- to three-fold increased risk compared to the general population [5]. This risk is multifactorial. The hypercoagulable state created by the chronic inflammatory ongoing process in IBS is a novel theory explaining the hypercoagulable state. On the other hand, IBD also causes relative hyperfibrinolysis secondary to decreased fibrinolysis, which leads to further increased risk of thrombosis [6]. Other factors contributing to VTE in IBD include acquired thrombophilias like anticardiolipin as well as medications like steroids, cyclosporine, and anti-TNF [5]. The treatment of VTE in IBD follows the same pathway for the general population with more attention regarding thromboprophylaxis in case of hospital admission for an acute flare-up venous thrombosis is worse in IBD patients than in the general population [5]. Infliximab is a chimeric (human-murine) monoclonal antibody that antagonizes tumor necrosis factor (TNF). It blocks and neutralizes the effect of TNF and reduces the levels of pro-inflammatory mediators, which in turn help to treat Crohn’s disease and other autoimmune diseases [7]. It is only given via the intravenous route because the digestive system will destroy the drug [8]. This is pivotal given the enteropathy in IBD patients. The most common side effects are related to the immune system suppression like serious infections (fungal, bacterial, or viral), reactivation of tuberculosis and hepatitis, drug-induced lupus, and increase the risk for certain types of cancer like lymphoma [2]. Side effects not related to the immune system like thromboembolic are infrequent. The first two case reports identified the occurrence of pulmonary embolism during infliximab treatment were in 2001 and 2003, respectively [4, 9]. The mechanism by which infliximab contributes to the development of pulmonary embolism is still not very clear. Anti-DNA-antibodies and anticardiolipin antibodies were recognized to be associated with pulmonary embolism induced by infliximab [4]. it is debated whether infliximab by itself causes a hypercoagulable state in patients during treatment or the production of autoantibodies (anti-double-stranded DNA and anticardiolipin antibodies) is the main reason behind the development of VTE. Both cases had a higher titer of anti-DNA-antibodies and anticardiolipin antibodies during active treatment [9]. In our presented case, those antibodies were not assessed during this hospital encounter. Some argue for coagulopathy screening before initiating treatment with infliximab although no contraindications for the treatment even if the patient tested positive [5]. This case report's abstract was presented in the 2019 Annual Meeting of the Society of General Internal Medicine 2019 [10].

## Conclusions

This case describes an unusual occurrence of venous thromboembolism (VTE)/pulmonary embolism in a patient on infliximab therapy. Although infliximab is linked to multiple common side effects, we should be aware of its thromboembolic aptitude. It is theorized that autoantibodies (anti-double-stranded DNA and anticardiolipin antibodies) contribute to a hypercoagulable state, which is known to cause VTE. Given the possible implications in the clinical practice of patients with inflammatory bowel diseases who are at a higher risk for VTE, further awareness and investigations are warranted.
